# Retraction Note: Liposome co-incubation with cancer cells secreted exosomes (extracellular vesicles) with different proteins expressions and different uptake pathways

**DOI:** 10.1038/s41598-026-61285-0

**Published:** 2026-07-07

**Authors:** Sherif E. Emam, Hidenori Ando, Amr S. Abu Lila, Taro Shimizu, Keiichiro Okuhira, Yu Ishima, Mahmoud A. Mahdy, Fakhr-eldin S. Ghazy, Ikuko Sagawa, Tatsuhiro Ishida

**Affiliations:** 1 Department of Pharmacokinetics and Biopharmaceutics, Institute of Biomedical Sciences, Tokushima University, 1-78-1 Sho-Machi, Tokushima, 770-8505 Japan; 2 Department of Molecular Physical Pharmaceutics, Institute of Biomedical Sciences, Tokushima University, 1-78-1 Sho-Machi, Tokushima, 770-8505 Japan; 3 Department of Pharmaceutics and Industrial Pharmacy, Faculty of Pharmacy, Zagazig University, Zagazig, 44519 Egypt; 4 Support Center for Advanced Medical Sciences, Institute of Biomedical Sciences, Tokushima University, 1-78-1 Sho-Machi, Tokushima, 770-8505 Japan; 5 Department of Pharmaceutics, College of Pharmacy, Hail University, 81442 Hail, Saudi Arabia

Retraction of: *Scientific Reports* 10.1038/s41598-018-32861-w, published online 27 September 2018

The Editors have retracted this Article following an institutional investigation at Tokushima University.

Following publication of this Article, and its subsequent Correction, the investigation identified overlapping images in Figure 4, Supplementary Figures 3 and 4, as well as in Supplementary Figure 4 of the Correction. The Authors stated that the data from the time at the original experiment cannot be validated for correction and agreed to retract the Article. The Editors therefore have lost confidence in the data presented in this Article.

Tatsuhiro Ishida, Keiichiro Okuhira, Ikuko Sagawa, Sherif Emam, Amr Abu Lila, Yu Ishima and Hidenori Ando agree with this retraction. Mahmoud A. Mahdy and Fakhr-eldin S. Ghazy did not respond to correspondence regarding this retraction. Taro Shimizu could not be contacted by the Editors.

